# An Unusual and Rare Presentation of Pulmonary Aspergillosis: Endobronchial Aspergilloma

**DOI:** 10.1155/2021/5525858

**Published:** 2021-05-27

**Authors:** Mohammed Ali, Aisha Mujahid, Rahul Dadhwal, Abhay Vakil, Humayun Anjum, Salim Surani

**Affiliations:** ^1^Department of Pulmonary Medicine, Corpus Christi Medical Center, Corpus Christi, TX, USA; ^2^Shadan Institute of Medical Sciences, KNR University of Health Sciences, Telangana, Warangal, India; ^3^Department of Pulmonary, Critical Care and Sleep Medicine, Texas A&M University Health Science Center, College Station, TX, USA

## Abstract

Aspergillosis is a wide spectrum of the disease process that is caused by the fungus *Aspergillus*. Endobronchial aspergilloma is a very rare type of aspergillosis which is not yet included in the classification of aspergillosis. Due to its rare nature and a limited number of cases, there are no current treatment guidelines. Here we present the case of a 57-year-old female with an endobronchial aspergilloma. The patient was started on intravenous voriconazole and subsequently discharged on oral voriconazole.

## 1. Introduction

Aspergillosis refers to a disease caused by a fungus of the genus *Aspergillus*, with the most common species being *Aspergillus fumigatus* [1]. The most commonly affected organs are the lungs [2]. Pulmonary aspergillosis causes a spectrum of clinical syndromes: invasive pulmonary aspergillosis (IPA), chronic pulmonary aspergillosis (CPA), and allergic bronchopulmonary aspergillosis (ABPA) [1]. IPA is a devastating opportunistic infection that occurs mostly in severely immunocompromised patients such as those with hematopoietic stem cell transplant, prolonged neutropenia, advanced AIDS, or solid organ transplant patients. CPA is further classified into aspergilloma, *Aspergillus* nodules, chronic cavitary pulmonary aspergillosis (CCPA), chronic fibrosing pulmonary aspergillosis (CFPA), and subacute invasive aspergillosis previously known as chronic necrotizing pulmonary aspergillosis (CNPA) [3]. Aspergilloma is a mass more commonly known as a “fungus ball” consisting of fungal hyphae mixed with cellular debris and mucus. An aspergilloma usually forms within the cavities in the lungs. It may occur in a person with normal immunity but with structurally abnormal lungs, with preexisting cavities that lead to airflow stasis [2]. *Aspergillus* nodules are single or multiple nodules that form without a cavity. CCPA usually shows multiple cavitary lesions with pulmonary and/or systemic symptoms. These cavitary lesions may enlarge and coalesce forming larger cavities. If left untreated, CCPA usually progresses to CFPA which leads to extensive fibrosis with fibrotic destruction involving at least two lobes and leading to significant loss of lung function. Subacute invasive aspergillosis occurs in those with mildly immunocompromised states, most commonly alcoholics, malnourished patients, chronic steroids, etc. [4]. ABPA is a complex hypersensitivity reaction in response to the colonization of the airways with *Aspergillus* and occurs almost exclusively in asthma and cystic fibrosis patients. Endobronchial aspergilloma (EBA) is a rare presentation of pulmonary aspergillosis with only a handful of reported cases in the literature. Here, we present a case of endobronchial aspergilloma with significant space-occupying and obstructing mass.

## 2. Case Presentation

**Figure 1:**
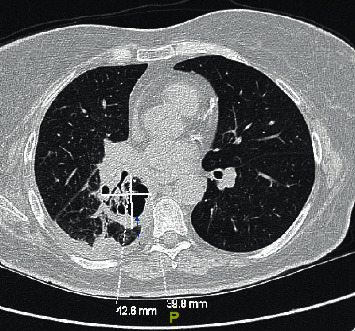
CT chest revealing the right upper lobe cavitary lesion (blue arrow).

**Figure 2:**
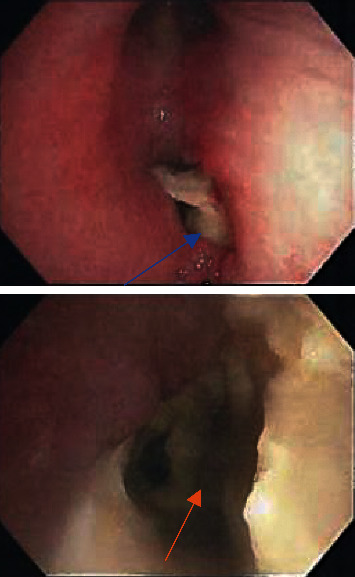
Solid obstructing endobronchial aspergilloma (blue arrow) in the right bronchus intermedius extending distally (orange arrow).

**Figure 3:**
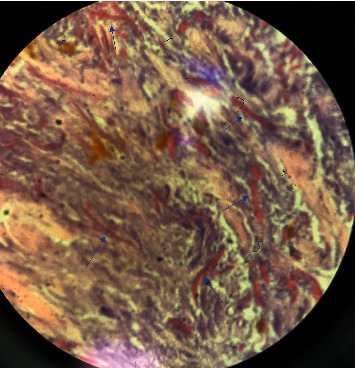
Biopsy of the endobronchial lesion with abundant septate hyphae (blue arrows).

This is a 57-year-old female with a recent history of thrombotic thrombocytopenic purpura that was treated with plasmapheresis and pertinent medical history of diabetes, COPD, and active smoker. She was evaluated in the pulmonary office for a cavitary lung lesion and was admitted for further workup including bronchoscopy. Laboratory results obtained during the admission were all within normal limits including a white blood cell count of 7.12 × 10^3^ with a normal differential and normal complete metabolic panel. Vital signs on admission were all within normal limits with a temperature of 98.9 degrees Fahrenheit, blood pressure of 126/76 mmHg, pulse 80 beats per minute, and respiratory rate of 11 breaths per minute. CT scan of the chest was obtained which did reveal a cavitary lesion in the right upper lobe (Figure 1) and atelectasis of the right middle and lower lobes. She then underwent bronchoscopy, which revealed an obstructing mass in the right bronchus intermedius with abnormal changes in the mucosa distal to the mass (Figure 2). A closer examination revealed some bleeding around the mass. Multiple biopsies were then obtained revealing abundant septate hyphae consistent with *Aspergillus* (Figure 3). A bronchoalveolar lavage (BAL) was performed and sent for culture which later grew *Klebsiella pneumoniae* and *Aspergillus fumigatus*. A fungal antibody profile was also obtained on BAL fluid which was positive for *Aspergillus fumigatus* (Table 1). *Aspergillus* galactomannan antigen test was also positive on BAL fluid. Beta-d-glucan test on BAL fluid was negative (Table 1). The patient was started intravenous voriconazole and subsequently switched to oral on discharge with plans for a repeat follow-up bronchoscopy in 6 months.

## 3. Discussion

*Aspergillus* is a fungus that exists in the environment, and it can be identified in organic matter. It exists in spore and hyphae form. The majority of illness in humans is caused by two species *Aspergillus fumigatus* and *Aspergillus niger* [1]. The most commonly affected organ in the body is the lungs; inhalation of the spores can cause pulmonary aspergillosis [1]. This is a rare occurrence and often occurs in immunocompromised patients, patients with underlying systemic illnesses, or patients with structural lung abnormalities.

As mentioned earlier, aspergillosis involves discrete syndromes: invasive aspergillosis, chronic pulmonary aspergillosis (CPA), and allergic bronchopulmonary aspergillosis (ABPA) [5]. Invasive aspergillosis is the most invasive on the spectrum and affects severely immunocompromised patients; it is the major cause of morbidity and mortality in that group of patients. CPA is locally invasive and presents in patients with chronic lung disease. ABPA is the most noninvasive of the three, and it is a hypersensitivity reaction to *Aspergillus*. An aspergilloma is a fungus ball, also called a mycetoma, that can form in a preexisting lung cavity. These cavities are usually formed due to some other illness or underlying pathology, for example, cystic fibrosis, treated tuberculosis, and emphysematous bullae [5]. Another separate type of pathology involving aspergillosis which can also exist is an endobronchial aspergilloma, it is a distinct entity and not classified with other aspergillosis, and there is also not much published about the disease process until very recently [6].

The most common culprit in EBA and human infection is *Aspergillus fumigatus* species [1]. The infectious cycle begins with the production of conidia (asexual spores). The organism is inhaled through the environment, most commonly decaying organic matter. It then becomes deposited in the bronchioles and alveoli. In healthy individuals, conidia are opsonized and then cleared by alveolar macrophages by phagocytosis and this process also promotes neutrophil recruitment to the site of inoculation [1, 4]. In some patients, conidia evade phagocytosis and can germinate. The development of invasive aspergillosis as well as EBA is based on host immunity. The primary risk factors for the development of EBA are immunodeficiencies and underlying lung disease. However, it may occur in a person with normal immunity but with structurally abnormal lungs or a person with chronic lung disease [7]. It is characterized by the massive intrabronchial growth of *Aspergillus fumigatus*. CNPA or ABPA may be associated with EBA. The most common underlying cause is tuberculosis. Although patients are asymptomatic, a few many experience cough, dyspnea, fever, weight loss, and malaise. EBA on X-ray and CT appears as a nonhomogenous mass with a classic air meniscus sign surrounding it. The mass is also somewhat mobile and can change position when the patient's position is changed. It is most often located in the upper lobes [5, 6]. EBA is a very rare entity and is typically encountered as an incidental finding on bronchoscopy [6]. An endobronchial mass the aspergilloma can be mistaken for lung cancer and can also sometimes hide underlying cancer. It can be very easily diagnosed with histopathologic examination of the endobronchial mass revealing the fungal elements.

A review of literature revealed only a handful of published cases and 1 case series reporting EBA. As mentioned recently by Ngu et al. in a comprehensive review, a total of 28 cases were thus far published involving discreet endobronchial aspergilloma [5]. There are no established treatment guidelines or recommendations for EBA. Since aspergillosis represents a spectrum of the disease, the treatment is also variable and individual specific [8]. Pulmonary aspergillosis with cavitary lesions in the parenchyma is treated surgically with the limited role of systemic antifungal therapy. However, EBA, the current consensus, seems to be a bronchoscopic intervention followed by systemic antifungal therapy [8]. Short-term follow-up bronchoscopy is recommended to evaluate for a response to therapy. EBA specifically is also occasionally treated with surgical resection to prevent life-threatening hemoptysis. Bronchial artery embolization is often used to prevent postop hemoptysis. The surgical option is considered for patients needing single lobe resection and having good lung function. Other indications for surgical resection include the inability to take azole therapy, resistance to azoles, or any other concurrent infections complicating the disease course. Pre- and postoperative antifungal therapy usually voriconazole is given to minimize the risk of postoperative pleural aspergillosis [9]. Antifungal therapy also provides some benefits for the treatment of a simple aspergilloma [10]. Asymptomatic patients, with stable radiographic findings over many months, may require no therapy, although monitoring for progression to chronic cavitary pulmonary aspergillosis and sometimes with extensive fibrosis can occur; therefore, close observation is required [8]. Spontaneous resolution of the aspergilloma occurs in fewer than 10 percent of the patients [5].

## 4. Conclusion

EBA is a rare presentation and is usually diagnosed incidentally during bronchoscopy. It can occur in immune-competent patients with underlying structural lung disease. Much remains to be learned as the optimal treatment and duration of medical therapy are not yet established. Recognition and initiation of therapy are paramount, and if left untreated, it progresses to cavitary lung disease and may progress to fibrosis.

## Figures and Tables

**Table 1:** Various pertinent test results.

Test	Result
White blood cell count	7.12 × 10^3^
BAL culture	*Klebsiella pneumoniae*
	*Aspergillus fumigatus*
BAL fungal antibody	*Aspergillus fumigatus*; positive
	*Aspergillus flavus*; negative
	*Aspergillus niger*; negative
	*Blastomyces*; negative
	*Coccidioides*; negative
	*Histoplasma*; negative
BAL *Aspergillus* galactomannan antigen	Positive
Beta-d-glucan	Negative
T-Spot TB test	Negative

## Data Availability

No data were used to support this study.

## References

[1] Kousha M., Tadi R., Soubani A. O. (2011). Pulmonary aspergillosis: a clinical review. *European Respiratory Review*.

[2] Kosmidis C., Denning D. W. (2014). The clinical spectrum of pulmonary aspergillosis. *Thorax*.

[3] Denning D. W., Cadranel J., Beigelman-Aubry C. (2015). Chronic pulmonary aspergillosis: rationale and clinical guidelines for diagnosis and management. *European Respiratory Journal*.

[4] Godet C., Philippe B., Laurent F., Cadranel J. (2014). Chronic pulmonary aspergillosis: an update on diagnosis and treatment. *Respiration*.

[5] Ngu S., Narula N., Abureesh M., Li J. J., Chalhoub M. (2019). Endobronchial aspergilloma-A comprehensive literature review with focus on diagnosis and treatment modalities. *European Journal of Clinical Microbiology & Infectious Diseases*.

[6] Huang D., Li B., Chu H. (2017). Endobronchial aspergilloma: a case report and literature review. *Experimental and Therapeutic Medicine*.

[7] Soubani A. O., Chandrasekar P. H. (2002). The clinical spectrum of pulmonary aspergillosis. *Chest*.

[8] Patterson K. C., Strek M. E. (2014). Diagnosis and treatment of pulmonary aspergillosis syndromes. *Chest*.

[9] Ali M., Mujahid A., Vakil A., Anjum H., Surani S. (2020). Solid endobronchial aspergilloma: a rare entity. *Chest*.

[10] Hirano T., Yamada M., Igusa R. (2016). Two cases of endobronchial aspergilloma complicated with primary and metastatic lung cancer: a case report and literature review. *Respiratory Investigation*.

